# Supplementary material for: Validation of models using basic parameters to differentiate intestinal tuberculosis from Crohn’s disease: A multicenter study from Asia
Source: PLoS One. 2020 Nov 30;15(11):e0242879. doi: 10.1371/journal.pone.0242879 (PMC7703980; doi:10.1371/journal.pone.0242879)
Supplement: S1 Table — (DOCX) [file pone.0242879.s001.docx]

**S1 Table.** Validation of the score by Lee, *et al*. in our total cohort of 530 patients

| For diagnosis of intestinal tuberculosis | Total cohort  (N=530) | Thai cohort  (n=241) | Hong Kong cohort  (n=289) |
| --- | --- | --- | --- |
| **Obtained a score unequal to “0”** | 387 (73.0%) | 195 (81.0%) | 192 (66.5%) |
| **Sensitivity** | 96% | 96% | 96% |
| **Specificity** | 47% | 33% | 56% |
| **Accuracy** | 237/387 (61.2%) | 120/195 (61.5%) | 117/192 (60.9%) |
| **Indeterminate diagnosis**  **(obtained a “0” score)** | 143 (27.0%) | 46 (19.0%) | 97 (33.5%) |
