# Supplementary material for: Validation of models using basic parameters to differentiate intestinal tuberculosis from Crohn’s disease: A multicenter study from Asia
Source: PLoS One. 2020 Nov 30;15(11):e0242879. doi: 10.1371/journal.pone.0242879 (PMC7703980; doi:10.1371/journal.pone.0242879)
Supplement: S2 Table — (DOCX) [file pone.0242879.s002.docx]

**S2 Table.** Models integrating computed tomography enterography (CTE)

| **Authors** | **Country** | **Study design** | **Model type** | **Parameters** | **Model detail** | | **Performance** |
| --- | --- | --- | --- | --- | --- | --- | --- |
| Zhao HS, *et al*.  Inflamm Bowel Dis 2014 | China | Retrospective,  CD 141, ITB 47 | LR model | 6 clinical  and 8 CTE findings | Clinical model  Hematochezia  Perianal disease  PPD test  Ascites  Pulmonary TB  Night sweats | CTE model  Left colon  Asymmetrical wall  Abscess  Comb sign  LN along right colic artery  Contracted IC  Fixed patulous IC  LN with necrosis | AuROC  Clinical model: 0.916  CTE model: 0.986 |
| Kedia S, *et al*.  Indian J Gastroenterol 2015 | India | Retrospective,  CD 54, ITB 50 | Scoring system | 3 CT/CTE findings | Long segment involvement +  (1–ileocecal region involvement) +  (1–LN ≥1 cm) | | Risk score for CD  3: Sen. 37, Spec. 90  Risk score for ITB  0: Sen. 14, Spec. 100 |
| Mao R, *et al*.  Endoscopy 2015 | China | Prospective, consecutive  67 CD, 38 ITB for training;  40 CD, 20 ITB for validation | Algorithm (combining Lee’s endoscopic score) | 2 CTE findings and  8 endoscopic findings | Presence of comb sign and/or  segmental small bowel lesion | | Increased accuracy of endoscopic score alone  71.6% - >88.3% |
| Kedia S, *et al*.  J Gastroenterol Hepatol 2018 | India | Retrospective,  32 CD, 27 ITB for training;  38 CD, 31 ITB for validation | Scoring system | 2 CT/CTE findings | VF/SC ratio >0.63 +  long segment involvement | | Validation set:  Risk score for CD  2: Sen. 50, Spec. 97  Risk score for ITB  0: Sen. 61, Spec. 84 |

**Abbreviations:** AuROC, area under receiver operating characteristic curve; CD, Crohn’s disease; CTE, computed tomography enterography; IC, ileocecal valve; LN, lymph node; LR, logistic regression; PPD, purified protein derivative; Sen, sensitivity; Spec, specificity; TB, tuberculosis; VF/SC ratio, visceral fat to subcutaneous fat ratio
