# Supplementary material for: Validation of models using basic parameters to differentiate intestinal tuberculosis from Crohn’s disease: A multicenter study from Asia
Source: PLoS One. 2020 Nov 30;15(11):e0242879. doi: 10.1371/journal.pone.0242879 (PMC7703980; doi:10.1371/journal.pone.0242879)
Supplement: S3 Table — (DOCX) [file pone.0242879.s003.docx]

**S3 Table.** Models integrating clinical, endoscopic, pathology, imaging, and laboratory findings

| **Authors** | **Country** | **Study design** | **Model type** | **Parameters** | **Model detail** | | **Performance** |
| --- | --- | --- | --- | --- | --- | --- | --- |
| Huang X, *et al*.  World J Gastroenterol 2015 | China | Prospective    CD 25, ITB 40 | Scoring system | 12 findings (2 clinical, 5 endoscopic, 4 CTE, and 1 IGRA) | Favors CD (+1)  Longitudinal ulcer  Nodular hyperplasia  Cobblestone appearance  Intestinal diseases  Intestinal fistulas  Target sign  Comb sign | Favors ITB (-1)  Night sweats  Positive PPD test  Positive T-SPOT.TB  Ring-shaped ulcer  Ulcer scars | AuROC: 0.997 |
| Bae JH, *et al*.  Inflamm Bowel Dis 2017 | Korea | Prospective    CD 40, ITB 40 for training;  CD 14, ITB 23 for validation | Scoring system | 8 endoscopic findings,  2 images (CXR, SBFT), and 2 laboratory tests (ASCA, IGRA) | Endoscopic score  (8 findings) | Lab-Radio score  Favors CD (+1)  - Proximal SB (SBFT)  - ASCA  Favors ITB (-1)  - Pulmonary TB (CXR)  - IGRA | AuROC  Training: 0.990  Validation: 0.981 |
|  |  |  |  |  | (+)🡪1, 0🡪0, (-)🡪(-1) | (+)🡪1, 0🡪0, (-)🡪(-1) |  |
|  |  |  |  |  | Summation  (-2, -1) => ITB, (0,1,2) => CD | |  |
| Wu X, *et al*.  Inflamm Bowel Dis 2018 | China | Prospective    CD 107, ITB 60 for training;  CD 46, ITB 26 for validation | LR model | 5 findings (2 clinical, 2 endoscopic, and 1 IGRA) | Perianal disease  Longitudinal ulcer  Left colon  Pulmonary TB  TB-specific Ag to phytohaemagglutinin | | AuROC  Training: 0.975  Validation: 0.950 |
| He Y, *et al*.  Am J Gastroenterol 2019 | China | Prospective    CD 143, ITB 69 for training;  CD 76, ITB 22 for validation | Step 1: Select variable from a random forest regression model  Step 2: LR model | 2 models  8 findings (1 clinical, 2 endoscopic, 3 CTE, and 2 IGRA/PPD) | Model 1  Age  Rectal involvement  Transverse ulcer  Skipped involvement of small bowel  Comb sign  IGRA | | AuROC  Training: 0.977  Validation (cut-off *p*=0.5)  Sen. 86.8%  Spec. 90.9%  Acc. 87.8% |
|  |  |  |  |  | Model 2  Age  Rectal involvement  Transverse ulcer  Skipped involvement of small bowel  Target sign  PPD | | AuROC  Training: 0.930  Validation (cut-off *p*=0.5)  Sen. 84.2%  Spec. 100%  Acc. 87.8% |
| Limsrivilai, *et al*.  Am J Gastroenterol 2017 | Thailand | Meta-analysis  Validation cohort 29 CD, 22 ITB | Step 1: select significant variables with low heterogeneity based on meta-analytic results  Step 2: integrate the variables into Bayesian model | 9 clinical, 8 endoscopic, 5 pathology, 5 CTE, and 1 IGRA  (can select only available parameters) | [bit.ly/ITBvsCD](file:///C:\Users\Jackie\Desktop\Julajak\bit.ly\ITBvsCD) | | AuROC  Clinical + endoscopy: 0.920  Clinical + endoscopy + pathology findings: 0.943 |

**Abbreviations:** ASCA, anti-*Saccharomyces cerevisiae* antibody; AuROC, area under receiver operating characteristic curve; CD, Crohn’s disease; CTE, computed tomography enterography; CXR, chest X-ray; IGRA, interferon gamma release assay; ITB, intestinal tuberculosis; LR, logistic regression; PPD, purified protein derivative; SBFT, small bowel follow through; SB, small bowel; Sen, sensitivity; Spec, specificity; Acc, accuracy; TB, tuberculosis; T-SPOT.TB, a type of ELISpot assay that is used for tuberculosis diagnosis that belongs to the group of interferon gamma release assays
