# Supplementary material for: Validation of models using basic parameters to differentiate intestinal tuberculosis from Crohn’s disease: A multicenter study from Asia
Source: PLoS One. 2020 Nov 30;15(11):e0242879. doi: 10.1371/journal.pone.0242879 (PMC7703980; doi:10.1371/journal.pone.0242879)
Supplement: Attachment — Submitted filename: Response to Reviewers.docx [file pone.0242879.s005.docx]

Dear Professor Pal Bela Szecsi,

We would like to thank the editor and reviewers for examining our manuscript entitled, “Validation of models using basic parameters to differentiate intestinal tuberculosis from Crohn’s disease: A multicenter study from Asia” (PONE-D-20-14222). We have reviewed each of the reviewers’ comments and provided a point-by-point response as below. We are submitting our revised manuscript for consideration and hope you find this version acceptable for publication in *PLOS ONE*.

Thank you very much

Yours sincerely,

Julajak Limsrivilai, M.D., MSc.

Choon Kin Lee, M.D.

Siew C Ng, M.D., Ph.D.

Reviewer #1: The study is interesting, largely well written and has a large data set. However, there are important methodological issues which might affect the validity of the findings of the study.
Major Concerns
*• It is surprising that only three models were chosen for evaluation. In a recent review (See Intestinal Res* [*https://doi.org/10.5217/ir.2019.09142*](https://doi.org/10.5217/ir.2019.09142)*), there are a large number of models which have been reported. How did the authors choose these three models and ignore others. Also, radiology has a very important role and exclusion of radiological models is concerning.*

 Response:       Thank you for the positive comments and for raising an important issue. We appreciate that several other models have been reported which included cross-sectional imaging and serology. Owing to the retrospective and real life study design of this paper, not all subjects had undergone cross sectional imaging. Most of the imaging studies in our cohort were conventional CT abdomen and not CT enterography. Since the majority of the previous studies had used CT findings obtained from CT enterography for their models, extrapolating some of these findings to other models might not be accurate. We have included these points in the limitations under discussion (page 19, line 351-358). In addition, the models using only clinical, endoscopic, and pathologic findings have an advantage that these are standard investigations for the diagnosis of Crohn’s disease or ITB in all countries including resource limited countries hence additional cost for investigations including interferon gamma release assay or cross sectional imagings are not required. Validation of their performance is also warranted.

We have included details on how we selected these four models (Lee et al., Makharia et al., Jung et al., Limsrivilai et al.) in the Methods section and the section on model validation (page 6, line 113-118).  We have also included under limitations that further prospective studies aiming to validate other models which incorporated radiological imaging will be necessary and this will form our future study.

*• It is surprising that the authors give an impression that the model is validated in a prospective manner. See the wording in abstract “newly diagnosed ITB and CD patients”. It is in the methods that it becomes clear that this data has been validated in a retrospective cohort. The word retrospective should be mentioned in the abstract*

Response: Thank you for the comments. We have added the word “retrospectively” in the abstract to better reflect our cohort.

*• The major concern about this study is that this is a validation study on a retrospective cohort. Since a number of parameters would not be recorded the performance of a particular algorithm/model will not be truly evaluated. This is a single factor which compromises to a great extent the findings of the study.*

Response: Thank you very much for the comments. We agree with the reviewer and have included this in the limitations of this study on page 18-19, line 338-346.

*• Also, in retrospective studies the lack of mention of a particular finding may be taken as absence of that finding. Eg. An endoscopist may ignore or not mention cobble-stoning unless the preformat mentions that this findings be reported OR endoscopist may ignore small/apthous ulcers when larger ulcers are present. This is a problem inherent in retrospective studies and therefore compromises the validity of findings.*

Response: Thank you very much for the comments. We have attempted to minimize this limitation by performing a review of pictures of endoscopic findings and specimen slides of pathologic findings by gastroenterologists and pathologist, respectively whereby an initial definitive diagnosis could not be made. For endoscopic findings, all available pictures of endoscopic findings of each patient were reviewed. For pathologic findings, 199 patients had slide specimens for review. The specimen slides of each patient were retrieved and reviewed by gastrointestinal pathologists. If the stain had faded out, resulting in unclear images, repeat staining was performed on the slides. We have added this information in the Methods section on page 6, line 107-111 and also emphasize this limitation in the discussion on page 18-19, line 338-346.

*• In some of the figures numbers don’t match: See night sweats, EIM in Table 2 ; Weight loss there is a gross mismatch. This gives an impression of carelessness in manuscript preparation and leaves the reviewer unsure whether to trust these findings*

Response: We appreciate the reviewer for the careful review and comments. We have corrected the typos and also rechecked other findings for accuracy.

Minor Concerns
*• It is incorrect to report that none of the models has been externally validated. The Limsrivilai model was created on basis of a Bayesian methodology using all published literature. This was then validated in a single center which is an external validatation (outside the dataset from which it was derived).*

Response: Thank you for the comments. We have revised the sentence “Models developed to distinguish Crohn’s disease (CD) from intestinal tuberculosis (ITB) have not been externally validated.” to “ Data on external validation of models developed to distinguish Crohn’s disease (CD) from intestinal tuberculosis (ITB) are limited.” in the abstract, and the sentence “However, none of these models have been externally validated and compared with the other models” to “However, the number of studies performed to externally validate those models are limited” in the first paragraph on page 4, line 73-74.

*• What is the definition of clinical response for Crohn disease. Was CDAI used or were endoscopies done and SES-CD calculated*

Response: Clinical response was defined based on physicians’ notes in medical records that there was improvement of abdominal symptoms (such as abdominal pain, diarrhea, and bloody stool) and general well-being, in combination with the improvement of inflammatory biomarkers. As data in this study were collected retrospectively, neither CDAI nor SES-CD was routinely recorded. The duration of at least six months of follow up was needed to confirm the clinical response. We have added this detail in the Methods section on page 5, line 98-101.

*• One of the models also takes into account the underlying prevalence of ITB or CD. What was the prevalence chosen. One believes that since it is a cross country study the prevalence would differ*

Response: Thank you for the comments, We used 0.28 for the prevalence of ITB and applied it to the ITB vs CD model. This number is based on the total number of patients in our cohort (427 CD and 163 ITB). We have added this detail in the Methods section on page 7, line 128-131.

*• How was response to ATT defined: was it endoscopic or clinical and what was the timing of response*.

Response: Among patients with negative results for the diagnosis of ITB, a diagnosis of ITB was based on empirical response to ATT. In this case, the response to ATT was defined as those having both a clinical and endoscopic response. A follow up endoscopy was performed at 2 to 6 months after initiation of treatment. This has been included in the methods section in page 6, line 105-106.

*Reviewer #2: This study looks at predictive models to differentiate between ITB and CD and looks at retrospective data sets to identify their reproducibility. It is a two center study and looks at three predictive models.*

Response: We thank the reviewer for the comments and summary of our paper. However, we do not see any suggestions. Please let us know if we miss something.

Journal Requirements:

When submitting your revision, we need you to address these additional requirements.

*1. Please ensure that your manuscript meets PLOS ONE's style requirements, including those for file naming. The PLOS ONE style templates can be found at*

[*https://journals.plos.org/plosone/s/file?id=wjVg/PLOSOne_formatting_sample_main_body.pdf*](https://journals.plos.org/plosone/s/file?id=wjVg/PLOSOne_formatting_sample_main_body.pdf) *and*

[*https://journals.plos.org/plosone/s/file?id=ba62/PLOSOne_formatting_sample_title_authors_affiliations.pdf*](https://journals.plos.org/plosone/s/file?id=ba62/PLOSOne_formatting_sample_title_authors_affiliations.pdf)

Response: We have corrected the format following according to the requirements.

*2. Please provide the date range when the medical data was originally collected, as well as the date(s) you accessed the data.*

Response: We have included the required information in the Methods section as follow;

“This multicenter retrospective cohort study included adult patients aged >18 years with a diagnosis of CD or ITB who were diagnosed and treated at four major GI centers in Thailand (Siriraj Hospital, King Chulalongkorn Memorial Hospital, Songklanakarin Hospital, or Phramongkutklao Hospital) from September 2004 to November 2018 and one major GI center in Hong Kong (Prince of Wales Hospital, The Chinese University of Hong Kong) from January 2000 to November 2016. The data was collected in all center between March 2017 and April 2019.”

*3. PLOS requires an ORCID iD for the corresponding author in Editorial Manager on papers submitted after December 6th, 2016. Please ensure that you have an ORCID iD and that it is validated in Editorial Manager. To do this, go to ‘Update my Information’ (in the upper left-hand corner of the main menu), and click on the Fetch/Validate link next to the ORCID field. This will take you to the ORCID site and allow you to create a new iD or authenticate a pre-existing iD in Editorial Manager. Please see the following video for instructions on linking an ORCID iD to your Editorial Manager account:* [*https://www.youtube.com/watch?v=_xcclfuvtxQ*](https://www.youtube.com/watch?v=_xcclfuvtxQ)

Response: We have linked up the corresponding author’s ORCID ID with this journal.

*4. Please include captions for your Supporting Information files at the end of your manuscript, and update any in-text citations to match accordingly. Please see our Supporting Information guidelines for more information:* [*http://journals.plos.org/plosone/s/supporting-information*](http://journals.plos.org/plosone/s/supporting-information)*.*

Response: This has been done.
