# Supplementary material for: Validation of models using basic parameters to differentiate intestinal tuberculosis from Crohn’s disease: A multicenter study from Asia
Source: PLoS One. 2020 Nov 30;15(11):e0242879. doi: 10.1371/journal.pone.0242879 (PMC7703980; doi:10.1371/journal.pone.0242879)
Supplement: Attachment — Submitted filename: Response to Reviewers.docx [file pone.0242879.s006.docx]

Dear Professor Pal Bela Szecsi,

We would like to thank the editor and reviewers again for examining our first revised manuscript entitled, “Validation of models using basic parameters to differentiate intestinal tuberculosis from Crohn’s disease: A multicenter study from Asia” (PONE-D-20-14222R1). We have reviewed each of the reviewers’ comments and provided a point-by-point response as below. We are submitting our revised manuscript for consideration and hope you find this version acceptable for publication in *PLOS ONE*.

Thank you very much

Yours sincerely,

Julajak Limsrivilai, M.D., MSc.

Choon Kin Lee, M.D.

Siew C Ng, M.D., Ph.D.

*Reviewer #2: The comments have addressed all points. I have no further comments. The authors have addressed all limitations.*

Thank you very much.

*Reviewer #3: The authors have done an interesting study comparing the existent models on differentiating CD and ITB. Though well presented, there are certain queries that need to be resolved-
1. The authors mention that they have compared the models which have utilized the only clinical, endoscopic and pathologic features so as to make it applicable in resource constraint countries. However, there are two other models by Yu et al (Digestion 2012;85:202-209.) and Li et al (Dig Dis Sci 2011;56:188-196.) which the authors have not included. Authors should discuss why these were excluded*

Thank you for raising this important issue. We were aware of these two models when we designed this study; however, we decided not to include them. The model by Yu *et al.* integrated the presence of granuloma without specific characteristics, making it difficult to differentiate whether the granuloma was related to CD or ITB. We also excluded the model by Li *et al.* because of two reasons. First, this model integrated the presence of rodent-like ulcer without specific detail, and thus the interpretation was unclear. The other reason was the use of tuberculin skin test (TST) as one of the indicators for the diagnosis of ITB. Many patients in our cohort received BCG vaccine at birth, and it could cause a false positive tuberculin skin test. Therefore, physicians generally did not do TST in this setting. None of the patients in our cohort had this data available. We have added this information in the section Materials and methods, subsection Model validation on page 6-7, line 124-133.

*2. One of these models (Li et al) includes tuberculin skin test which is easily available in resource constraint countries as well. Did the authors have data on TST in their cohort? If yes, then including this would add to the study.*

We agree that if tuberculin skin tests were available, it might have helped to distinguish ITB from CD. However, there is a concern about using TST in our patients as we mentioned above.

*3. The endoscopic and pathologic slides were reviewed by Gastroenterologist and Pathologists. Was central reading done or was it reviewed at different centers?*
The endoscopic findings were reviewed by experts of each center. The available pathologic slides were sent and reviewed by two experts from two centers. We added this details in the methods section on page 6 line 107-110. We recognized interobserver disagreement, and thus stated this issue in the limitations of this study. We have added that using central readers could minimize the variation in the interpretation in the limitations of study on page 19, line 363 as well.

*4. 29 of 147 ITB patients (20%) only had indeterminate diagnosis of ITB and rest had a definite diagnosis. Given the poor sensitivity of current diagnostic techniques for ITB, 80% definite diagnosis rate for ITB is rather surprising.*
We agree with the reviewer and the detailed information about the diagnosis of ITB in this study is provided in the answer to question#5. The numbers of ITB patients whose diagnosis required response to empirical antituberculosis agents in this cohort is lower than we expected as well.

*5. Authors should mention the proportion of patients who satisfied the different diagnostic criteria for ITB (e.g. positive AFB, culture, caseating necrosis, etc.)*
Among 147 ITB patients, 70 (47.6%) patients had pathological findings found either AFB or caseous granuloma, 35 (23.8%) patients had tissue culture growing mycobacterium tuberculosis, 36 (24.5%) patients had proven tuberculosis elsewhere, and 29 (19.7%) patients were diagnosed based on response to empirical antituberculosis therapy. We have added this information in the first paragraph of the results section on page 9 line 171-175.

*6. Similarly among patients with CD, how many received ATT trial and how many were diagnosed upfront?*

Nineteen (5%) of Crohn’s disease patients had received anti-tuberculous therapy without response before the diagnosis of Crohn’s disease was made. We have added this information in the first paragraph of the results section on page 9 line 175-177. This proportion is quite low. The reason could be that this study was conducted in tertiary-referral centers where some physicians might have experience with Crohn’s disease more than general physicians, resulting in more confident to treat Crohn’s disease when all TB tests were negative.

*7. Among the comparison of pathologic findings, authors should also mention about the prevalence of caseation necrosis in either group.*

The prevalence of caseation in each group was not presented in the table 2 because the caseous necrosis was present only in patients with ITB.

*8. The real value of these models lies in predicting the diagnosis of ITB in patients with indeterminate TB. With only 29 such patients the study becomes grossly limited in its aim of external validation of these models. This point should be clearly highlighted in discussion.*

Thank you for pointing out this issue. We do agree that the value of these models are limited to those with indeterminate diagnosis of ITB, which was quite a small number in this study, and we have added this limitation on page 20 line 373-376. Nonetheless, all diagnostic models were derived from patients with both indeterminate and definite ITB, and thus we opted to validate these models with similar patient population.
